# The efficacy of short tandem repeat polymorphisms versus single-nucleotide polymorphisms for resolving population structure

**DOI:** 10.1186/1471-2156-6-S1-S84

**Published:** 2005-12-30

**Authors:** John SK Kauwe, Sarah Bertelsen, Laura Jean Bierut, Gerald Dunn, Anthony L Hinrichs, Carol H Jin, Brian K Suarez

**Affiliations:** 1 Department of Psychiatry, Washington University of Medicine, St. Louis, MO, USA; 2 Department of Genetics, Washington University of Medicine, St. Louis, MO, USA

## Abstract

Accurately resolving population structure in a sample is important for both linkage and association studies. In this study we investigated the power of single-nucleotide polymorphisms (SNPs) in detecting population structure in a sample of 286 unrelated individuals. We varied the number of SNPs to determine how many are required to approach the degree of resolution obtained with the Collaborative Study on the Genetics of Alcoholism (COGA) short tandem repeat polymorphisms (STRPs). In addition, we selected SNPs with varying minor allele frequencies (MAFs) to determine whether low or high frequency SNPs are more efficient in resolving population structure. We conclude that a set of at least 100 evenly spaced SNPs with MAFs of 40–50% is required to resolve population structure in this dataset. If SNPs with lower MAFs are used, then more than 250 SNPs may be required to obtain reliable results.

## Background

Accurately resolving population structure in a sample is important for both linkage and association studies. Understanding population structure can allow us to use homogenous study groups, thus improving our ability to detect population specific linkage and ensuring that false linkage is not detected because of erroneously assigned allele frequencies. In association studies, differences in population structure between cases and controls can result in high rates of both type I and type II errors [e.g., [1-3]]. When population structure can be resolved, matching between cases and controls can be achieved and one possible confounding factor can be removed.

Short tandem repeat polymorphisms (STRPs) are highly variable markers that have proven to be very useful in resolving population structure. However, single-nucleotide polymorphism (SNP) assays are efficient and inexpensive, and the use of SNPs has become widespread. The resolving power of a set of SNPs will depend upon both the density of the markers and their frequencies. SNPs with minor allele frequencies (MAFs) of near 0.50 are assumed to be more ancient, while SNPs with low MAFs are assumed to be much more recent [4]. One hypothesis is that those SNPs with high MAFs predate the origins of modern human races and carry little useful information about population structure. It follows that SNPs with low MAFs, being much more recent polymorphisms, may be more informative in resolving population structure. Alternatively, the low heterozygosity of these SNPs may limit their usefulness (since the allele frequency differences between two populations would perforce be low); in this case, SNPs with high MAFs will be far more informative.

In this study we investigate the power of SNPs in detecting population substructure using results from STRPs as the gold standard. We first investigate the number of SNPs that are required to obtain results comparable to those of STRPs. Second, we determine whether lower or higher frequency SNPs provide more information regarding population structure.

## Methods

### Sample

This study includes 286 unrelated individuals from the Collaborative Study on the Genetics of Alcoholism (COGA) pedigrees [5]. We preferentially selected founders; however, in cases where 2 founders had large amounts of missing data, we selected the offspring for whom the most data was available. The self-reported race of these 286 individuals was as follows: 245 European Americans, 26 African Americans, 12 European American/Hispanics, and 3 African American/Hispanics. Each of these individuals was genotyped for the 328 STRPs from COGA, 4,720 SNPs from the Illumina linkage panel, and 11,120 SNPs from the Affymetrix mapping array which were prepared for Genetic Analysis Workshop 14 [5].

### Analyses

SNPs from each dataset were ranked according to MAF. To construct the Illumina and Affymetrix "low frequency" SNPs, we made groups starting with the lowest frequency SNP and progressively included the next 10 SNPs. The final groups included the 1,000 SNPs with the lowest MAFs in each dataset. For the "high frequency" SNPs, we began at the SNP with the highest MAF and constructed groups in the same manner. The MAFs of the SNPs in the four 1,000 SNP sets are described in Table 1.

Each of these groups was then analyzed with the computer program STRUCTURE to identify possible sub-structure in the sample of 286 unrelated individuals [6]. This method assumes that the sample contains a mixture of subpopulations and that within each subpopulation there is Hardy-Weinberg and linkage equilibrium between markers. This program identifies subpopulations of individuals who are genetically similar through a Markov chain Monte Carlo (MCMC) sampling procedure [7]. The STRP and SNP sets were run for 10,000 iterations after 10,000 burn-in replicates assuming a two-cluster solution. Although we tested various numbers of clusters, the solutions involving more than two clusters merely divided the European Americans into subgroups; for maximum parsimony, we used only the two cluster solution. The STRP results were used as a gold standard for comparison.

To compare the assignment probabilities of each individual between the SNPs and STRPs we took the absolute value of the differences between the probability generated by each set of SNPs and the one generated by the STRPs in each individual averaged across the individuals genotyped in both sets. We subtracted that number from one to obtain a measure of percent similarity between the SNP and STRP results. For example, assume that the probability of membership in groups 1 and 2 for a single individual in a SNP run is completely ambiguous (0.5 and 0.5, respectively). When this is compared with STRP probabilities of approximately 1 and 0; percent similarity for that individual would be calculated as, 1 - |1 - 0.5| = 0.5, or 50% similarity. This score implies a completely ambiguous assignment versus an absolute assignment and is the lowest expected score.

We also compared the frequency of SNPs in the self-reported European and African American groups. First the SNPs were sorted by the percentage of heterozygotes present in the European American individuals. Then we took the squared difference of the allele frequency of each group for each SNP in both the Illumina and Affymetrix datasets; this value is considered a measure of information content for each SNP. By this measure, a SNP that is fixed for one allele in European Americans and the other allele in African Americans would have an information content of 1.

## Results

**Figure 1:**
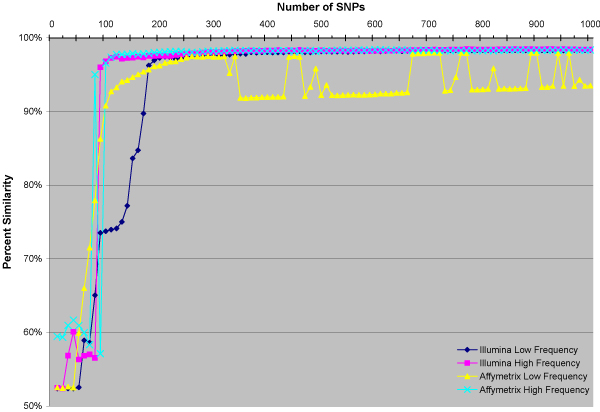
**Structure results of sets of SNPs compared to STRPs. **Similarity is one minus the absolute value of the differences between the probability generated by each set of SNPs and the one generated by the STRPs in each individual averaged across the individuals genotyped in both sets.

The results of the STRP and 1,000 SNP runs in STRUCTURE were in concordance with self-reported race for all the European Americans and African Americans (Table 2). The results of comparing each SNP set with the STRPs can be seen in Figure 1. For sets with less than 100 SNPs the classifications were unreliable. In both the Affymetrix and Illumina high frequency SNPs, sets with more than 100 SNPs resolved the population structure, yielding results that were essentially identical to those of the STRPs. However, for the low frequency SNPs, greater than 250 SNPs were required before giving results comparable to those of the STRPs.

In the case of the Affymetrix low frequency SNPs, the results do converge on that of the STRPs, but the solution appears to be multimodal [6]. This artifact may be due to the extremely low MAFs in that set (Table 1).

**Figure 2:**
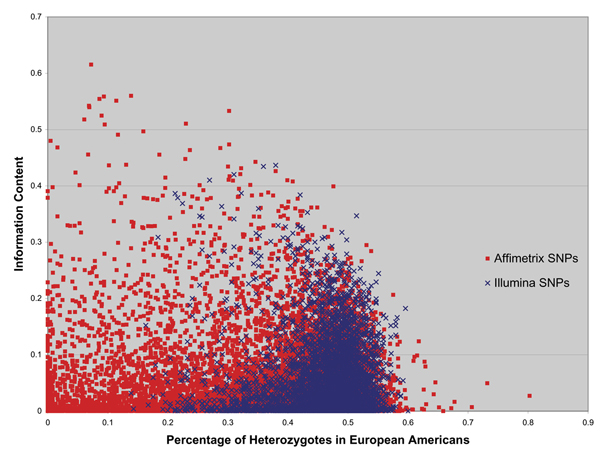
**Information content of each SNP. **Information content of each SNP is measured as the squared difference between the MAF in self-reported African American and European American individuals.

The distribution of information content in the SNPs is shown in Figure 2. We performed regression of information content on percent heterozygotes for the Affymetrix and the Illumina SNPs in Figure 2. The slopes of these lines are not significantly different from 0 and the intercepts are 0.053 and 0.051 for Affymetrix and Illumina, respectively. Although nearly all of the Illumina SNPs have between 40 and 50% heterozygotes, and the Affymetrix panel includes a large number of SNPs with low heterozygosity, it appears that by this measure a SNP chosen at random from either dataset would have approximately the same degree of informativeness.

## Discussion

The results in Figure 1 support the findings of a previous study that suggests that approximately 100 SNPs are required to resolve population structure in a sample [8]. However, these data also suggest that when SNPs with low MAFs are used, greater than 250 are required to obtain reliable results. We hypothesized that the SNPs with MAFs of near 0.50 may be much older than the origins of modern human races and therefore less informative than lower frequency SNPs that may have appeared closer to the time that modern *Homo sapiens *left Africa. However, these data suggest that on average high frequency SNPs provide better information. In fact, fewer SNPs of high frequency than low frequency are required to closely approach the STRP results. It appears that the potential greater sensitivity of more recent SNPs does not translate into an increase in useful information. It is also interesting to note that the similarity between the STRP and SNP runs never reaches 100%. The 96% similarity that is reached by these SNP sets is equivalent to an average difference in probability of just 4% per individual. This is likely due to the MCMC nature of STRUCTURE; multiple runs of the STRP dataset show a similar level of variation (even with 100,000 burn-in and 100,000 iterations).

By our measure of information content, low-frequency SNPs have the greatest potential to be informative. In fact, the 10 most informative SNPs have less than 20% heterozygotes in the European American sample. However, these low frequency SNPs also have great potential to provide information that is useless or misleading with regard to the ancestry of an individual. The vast majority of SNPs with a low percentage of heterozygotes provide little or no useful information. As a result, high frequency SNPs, on average, seem to provide better information. This is a likely explanation for why the results in Figure 1 show it requires far fewer SNPs of high MAF to resolve population structure effectively in this sample.

In these analyses we have identified a set of highly informative SNPs. Previous studies have shown that similar sets of SNPs have been effective at verifying self-reported ethnicity in other samples [9,10]. The SNPs we have identified may serve as a "genomic control set" in these data. Runs using the 20 SNPs with the highest differences in allele frequencies between populations show 97% similarity to the STRP results (Table 3). Future studies could confirm the general applicability of these SNPs by replicating these results in other samples.

## Conclusion

These data suggest that SNPs are a cost effective and informative replacement for STRPs when used to detect population structure. Based on these results, a set of approximately 100 SNPs with MAFs of 40–50% can resolve population structure. If SNPs with lower MAFs are randomly chosen, then more than 250 SNPs may be required to obtain reliable results. However, these results identify a subset of 20 SNPs that also reliably resolve population structure in this sample. These results suggest that a small "genomic control subset" selected based on allele frequency differences in the two populations could be quite useful. Although the regression indicates that any SNP, on average, shows a 5% allele frequency difference between the two populations, our results show that the SNPs with higher MAF are more useful for the STRUCTURE analyses. Thus, it is better, in general, to choose a SNP with MAF of 0.45 and 0.5 in Caucasians and African Americans than to choose a SNP with MAF of 0 and 0.05.

## Abbreviations

COGA: Collaborative Study on the Genetics of Alcoholism

MAF: Minor allele frequencies

MCMC: Markov chain Monte Carlo

SNP: Single-nucleotide polymorphisms

STRP: Short tandem repeat polymorphisms

## Authors' contributions

JSKK formatted the data files, analyzed the data, and drafted the manuscript. ALH participated in the data preparation and analysis and helped to draft the manuscript. SB, LJB, GD, CHJ, and BKS participated in the design and coordination of the study. All authors read and approved the final manuscript.

## Figures and Tables

**Table 1:** A description of MAFs and rates of missing data in the 1,000 SNP sets

Marker set	Mean MAF	SD	Range	Missing rate
Illumina Low Frequency	0.26	0.05	[0.08–0.33]	0.35%
Illumina High Frequency	0.48	0.01	[0.47–0.5]	0.19%
Affymetrix Low Frequency	0.04	0.02	[0–0.08]	5.44%
Affymetrix High Frequency	0.48	0.01	[0.46–0.5]	5.34%

**Table 2:** Probability of cluster membership based on the 328 COGA STRPs

		Average probability of membership in cluster	
		
Self reported race	*n*	European American	African American
European American	257	0.98	0.02
African American	29	0.08	0.92

**Table 3:** The 20 most informative SNPs, their information content (IC), and MAFs in the COGA GAW14 data

SNP ID	IC	MAF
rs2341823	0.62	0.16
rs719776	0.56	0.22
rs3843777	0.56	0.19
rs718387	0.55	0.15
rs723632	0.55	0.18
rs721684	0.54	0.13
rs2078588	0.54	0.15
rs1478785	0.53	0.33
rs1369290	0.52	0.16
rs1872861	0.52	0.13
rs719191	0.51	0.27
rs714857	0.51	0.14
rs2021781	0.50	0.19
rs526593	0.49	0.16
rs1438405	0.48	0.08
rs1371231	0.47	0.30
rs1352405	0.47	0.12
rs726391	0.47	0.39
rs725472	0.46	0.27
rs2351254	0.46	0.12
